# Key Ferroptosis Genes and their Predictive and Diagnostic Value in Fanconi Anemia

**DOI:** 10.33549/physiolres.935383

**Published:** 2025-04-01

**Authors:** Can MENG, Li HUANG, Hui HUANG, Zhenyu ZHAO, Xiangjun FU, Hongxia YAO, Bin WU

**Affiliations:** 1 Department of Hematology, Hainan General Hospital (Hainan Affiliated Hospital of Hainan Medical University), Hainan Province, P.R China; 2 Department of Sports Medicine, Hainan General Hospital, Hainan Province, P.R China

**Keywords:** Fanconi anemia, Ferroptosis, Gene, Diagnosis, Predictive

## Abstract

Fanconi anemia (FA) and ferroptosis both affect tumor-related processes. However, few studies have reported on genetic associations between FA and ferroptosis. Our study evaluated the usefulness of genes related to ferroptosis in predicting and diagnosing FA. Transcriptome sequencing data were collected from 11 normal participants and 21 patients with FA. Differential gene analysis, Kyoto Encyclopedia of Genes and Genomes (KEGG), Gene Ontology (GO) analysis, gene correlation analysis, protein-protein interaction network analysis, qRT-PCR, and pan-cancer analysis were performed. The pan-cancer analysis was carried out based on data obtained from the GTEx and TCGA databases. Two hundred ninety-eight differentially expressed genes were detected based on the comparison of FA patients and normal participants, among which four critical non-FA genes, *MAD2L1, ASPM, PCNA*, and *TOP2A*, were identified. Among the ferroptosis-related genes, five genes, including *CDKN1A, EMC2, FDFT1, HSPB1*, and *MT1G*, were identified as being associated with FA, and the areas under the curve (AUC) of these five ferroptosis-related genes were 0.907, 0.640, 0.902, 0.840, and 0.929, respectively. The AUC for the diagnosis of FA reached 1.000 when the five ferroptosis-related genes were used in combination. In addition, the expressions of *CDKN1A, EMC2, FDFT1*, and *HSPB1* were associated with the prognosis of multiple cancers (*P*<0.05). The five ferroptosis-related genes *CDKN1A, EMC2, FDFT1, HSPB1*, and *MT1G* exhibited excellent predictive effects for the diagnosis of FA.

## Introduction

Fanconi anemia (FA), a rare genetic disease, is characterized by birth defects, bone marrow failure (BMF), as well as a predisposition to malignancies, including leukemia and squamous cell carcinoma [1]. It is estimated that approximately 1 to 5 cases in 1 million births suffer from FA, 80 % of FA children and 90 % of older FA patients develop BMF [2]. Nearly 50 % of FA patients had myelodysplastic syndrome and/or acute myeloid leukemia (AML). The cancer risk of the head and neck, esophagus, gastrointestinal tract, vulva, and anus is approximately 50 times higher in FA patients [3,4]. Garaycoechea *et al*. [5] reported the tumor suppressor function of FA proteins in genome maintenance. Ferroptosis, a molecular process dependent on iron and oxidation, has been identified recently as a type of regulated cell death in cancerous and non-cancerous cells [6]. Death associated with the presence of iron promotes and inhibits tumor development, depending on releasing damage-associated molecular patterns in the microenvironments associated with the tumors and activating immune responses triggered by iron death injury [7].

Currently, abnormalities in 22 genes have been found to cause FA, including 18 clearly pathogenic genes (*FANCA, FANCB, FANCC, FANCD1/BRCA2, FANCD2, FANCE*, and others) and four suspected FA genes [8]. Together with a series of related regulatory proteins, these pathogenic genes, such as *MHF1, MHF2, FAAP20, FAAP24*, and *FAAP100*, constitute the FA/BRCA pathway [9]. It has been reported that the FA/BRCA pathway is critical in the process of DNA-ICL repair, and defects in this pathway can lead to cells in patients with FA that are highly sensitive to DNA cross-linking inducers, including mitomycin C (MMC), epoxybutane, and platinum compounds. Thus, patients with FA are more likely to develop various cancers. Recent studies have shown that the failure in normal bone marrow function in patients with FA may result from blocked hematopoietic stem cell (HSC) differentiation caused by the blocked FA/BRCA pathway repair of DNA-ICL [10].

Previous studies have shown that FA complementation group D2 (FANCD2) is protective against injuries caused by ferroptosis in bone marrow stromal cells (BMSCs). However, FA will cause FANCD2-deficiency, which increases lipid peroxidation in ferroptosis, resulting in BMSC injury [11]. BMSCs are an important component associated with the bone marrow hematopoietic microenvironment that could be related to the failure observed in bone marrow hematopoiesis caused by FA. Nevertheless, the patterns of expression and prognostic roles associated with these genes in FA remain unclear.

Due to recent advances in genomic sequencing technology, increased numbers of microarray analyses of datasets associated with diseases have been carried out. They have become ideal sources for investigating ferroptosis-related genes in various diseases. In this study, the PPI network was used to identify the critical genes *MAD2L1, ASPM, PCNA*, and *TOP2A* in FA, and correlation analysis was conducted using 16 iron death-related genes. Five genes, *CDKN1A, EMC2, FDFT1, HSPB1*, and *MT1G*, exhibited diagnostic value in FA. The mRNA levels of *CDKN1A, EMC2, FDFT1, HSPB*, and *MT1G*, were analyzed comprehensively, and any correlations between the levels of mRNA of these five genes and pan-cancer prognosis were identified.

## Methods

### Data source and study population

**Fig. 1:**
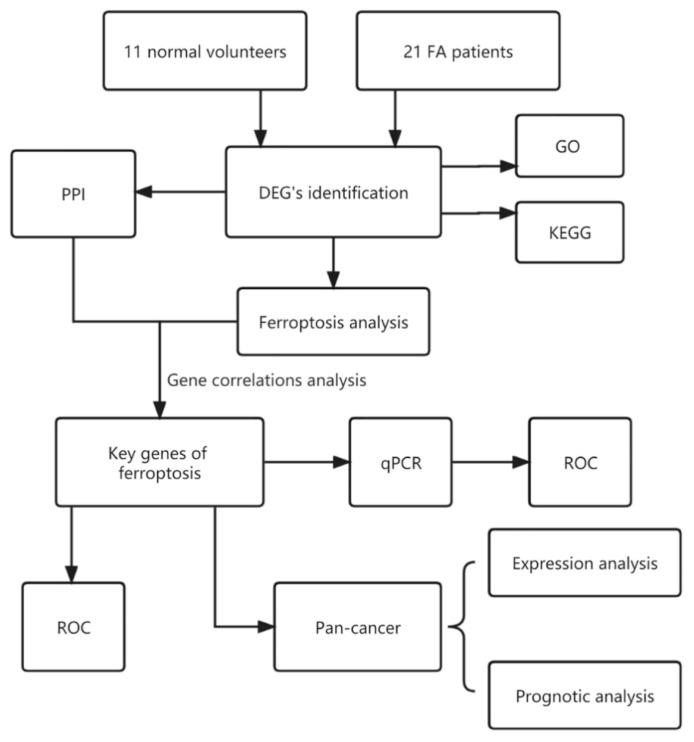
Flowchart of the bioinformatic analysis in the present study. FA, fanconi anemia. DEGs, differentially expressed genes. PPI, protein-protein interaction. ROC, receiver operating characteristic.

The GSE16334 dataset were extracted from the GEO database (https://www.ncbi.nlm.nih.gov/geo/). A total of 21 FA patients and 11 normal controls were included. Any FA patients presenting cytogenic evidence that indicated the existence of clonal evolution were excluded. In addition, any FA patient that exhibited acute leukemia was excluded. The RNA samples were prepared from low-density mononuclear cell fractions that had been freshly obtained. The design of the analysis protocol is shown in Fig. 1.

### Differential gene analysis

The R software Limma package (version: 3.40.2) was utilized to assess the differential mRNA expression. After adjustment, the P-value was examined to correct for the occurrence of any false positives in the GEO datasets. The “Adjusted *P* < 0.05 as well as the Log (Fold Change) >2.0 or Log (Fold Change) < −2.0” were used as the thresholds to screen for differential mRNA expression. A box plot was created using the ggplot2 package from the R software. The PCA graphs were drawn using the R good software package. The pheatmap package in the R software was used to develop the heatmap. The analytical methods mentioned above, as well as the R package, were obtained from the R foundation for statistical computing (2020) version 4.0.3.

### GO and KEGG analysis

Functional enrichment was used to analyze the data to confirm the underlying functions associated with the potential targets in this study. Gene Ontology (GO) is a commonly used program that annotates genes with known functions, including biological pathways (BP), molecular functions (MF), and cellular components (CC). An additional analytic tool used in this study was the Kyoto Encyclopedia of Genes and Genomes (KEGG) enrichment analysis, which serves as a resource to analyze gene functions as well as information related to high-level genome functions. Therefore, to increase our understanding of mRNA functions involved in this study, the ClusterProfiler package (version: 3.18.0) in R was utilized to determine the GO function of potential targets and assess the KEGG pathway enrichment. An adjusted *P*-value < 0.05 was considered statistically significant.

### Ferroptosis analysis

Genes associated with ferroptosis were identified based on the systematic analysis provided by Liu *et al*. [12] of the aberrant functions and additional functional implications associated with ferroptosis in cancer. The PCA graphs were created using the gord program in the R software package. The box plot was created using ggplot2 in the R software package. The analytical methods mentioned above, as well as the R package, were obtained from the R foundation for statistical computing (2020) version 4.0.3. A *P*-value < 0.05 was used to determine statistical significance.

### DEG Analysis using the Protein-Protein Interaction (PPI) Network

A DEG PPI network was developed using data obtained from the STRING database. Notably, the STRING database is a tool available online that can be used to analyze protein expression as well as protein interactions. These data can be utilized to acquire broad, unique, experimentally confirmed information that is predictive of various interactions. The combined score can be used to indicate how two proteins might interact. In this investigation, only pairs of interactions with a combined PPI score greater than 0.9 were considered to be significant. Molecular Complex Detection (MCODE), which is a Cytoscape plug-in, was used for the identification of any sub-clusters that occurred in the PPI network. The core gene associated with each sub-cluster was identified using the gene scores observed in the sub-clusters (MCODE score >8; number of nodes >8).

### Gene correlation analysis

Gene correlation analysis was used to identify any relationships that existed between the various genes. Spearman’s correlation analysis was then utilized to define the correlations among the quantitative variables that did not exhibit a normal distribution. A *P*-value less than 0.05 indicated statistical significance. Graphs that revealed multi-gene correlations were created using the pheatmap program found in the R software package [13–15].

### mRNAs validation using qRT-PCR

The Institutional Review Committee and the Ethics Committee of Hainan General Hospital approved this study, which met the overall medical ethics requirements (Approval No: Med-Eth-Re [2017] 01). Patients with FA were identified with diepoxybutane chromosomal breakage analysis or mitomycin C. Five FA patients and five normal volunteers were included in this analysis with the exclusion of any FA patients that exhibited cytogenetic evidence of clonal evolution. In addition, any FA patient that had acute leukemia also was excluded. The RNA was prepared using low-density mononuclear cell fractions that had been freshly acquired. Total RNA was extracted from previously frozen issues using beyozol (Beyotime Bio, Inc., China). RNA was reverse transcribed into cDNA utilizing the BeyoRT™ II cDNA Synthesis Kit (Beyotime Bio, Inc., China). Subsequently, 2 μg of cDNA was analyzed in each reaction with the BeyoFast™ SYBR Green One-Step qRT-PCR Kit (Beyotime Bio, Inc., Beijing, China). The analysis was carried out using the Applied Biosystems Real-Time PCR System (Applied Biosystems, Thermo Fisher Scientific, Waltham, MA, USA). Gene expression relative to the housekeeping gene *GAPDH* was determined using the 2-ΔΔCt method.

### Pan-cancer analysis

FA is a rare inherited syndrome associated with bone marrow failure that exhibits an extreme predisposition to the development of cancer, including breast invasive carcinoma (BRCA), glioblastoma multiforme (GBM), cervical squamous cell carcinoma (CSCC), head and neck squamous cell carcinoma (HNSC), ovarian serous cystadenocarcinoma (OV), AML, and brain lower grade glioma (LGG) [16,17]. Therefore, the tumors listed above were selected for inclusion in the pan-cancer analysis. The pan-cancer analysis performed in this investigation included expression analysis and prognostic analysis. The profiles for RNA-sequencing expression (level 3) and the corresponding clinical information for the tumors that are frequently triggered by FA were obtained from the GTEx and TCGA datasets. The methods used in the analyses and the R package were obtained from R version 4.0.3. Any P-values < 0.05 indicated statistical significance (*P < 0.05).

Expression analysis for two-group data was carried out using the Wilcox test. Prognostic analysis was carried out with univariate cox regression analysis. The resulting forest plot was developed using the ‘forestplot’ R package, and the forest indicated the *P*-value, HR, and 95 % CI for each variable.

### Statistical analysis

Continuous variables observed between the two groups were compared using either the Student’s t-test or the Mann–Whitney U-test when appropriate. The diagnostic value of expressed genes observed in FA patients was assessed using receiver operating characteristic (ROC) curves of univariate and joint models. Furthermore, the area under the curve (AUC) was utilized to determine the diagnostic value. The statistical analyses used in this study were carried out with R software (version 4.0.3) and SPSS version 19.0 software (SPSS, Inc., Chicago, IL, USA). A two-tailed *P*-value < 0.05 was used to determine statistical significance.

## Results

### Results of differential gene analysis

**Fig. 2:**
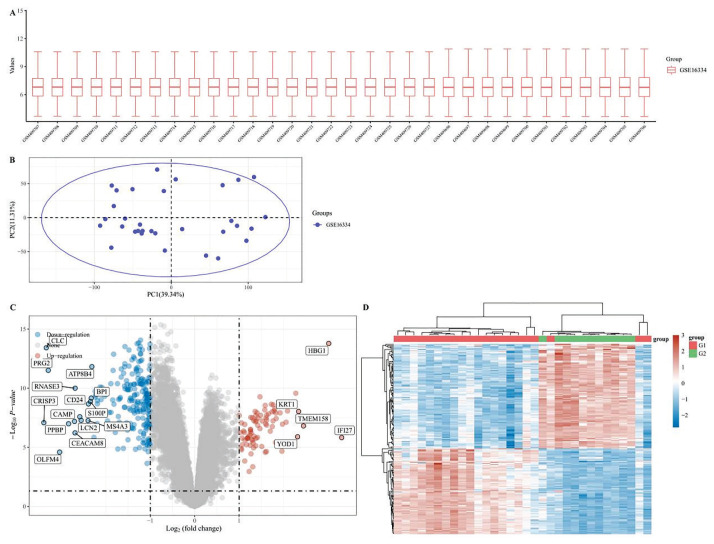
(**A**) Box plot after data standardization, different colors represent different data sets. (**B**) PCA results before batch removal for multiple data sets. (**C**) Volcano plots were constructed using fold-change values and adjusted P. The red point in the plot represents the over-expressed mRNAs and the blue point indicates the down-expressed mRNAs with statistical significance. (**D**) Hierarchical clustering analysis of mRNAs, which were differentially expressed between observation and normal group.

Two hundred ninety-eight genes were determined to be expressed differentially in the FA group in comparison to the normal group. Among these DEGs, 216 genes were identified as downregulated, and 82 were upregulated. The results are shown in Fig. 2.

### GO and KEGG results

**Fig. 3:**
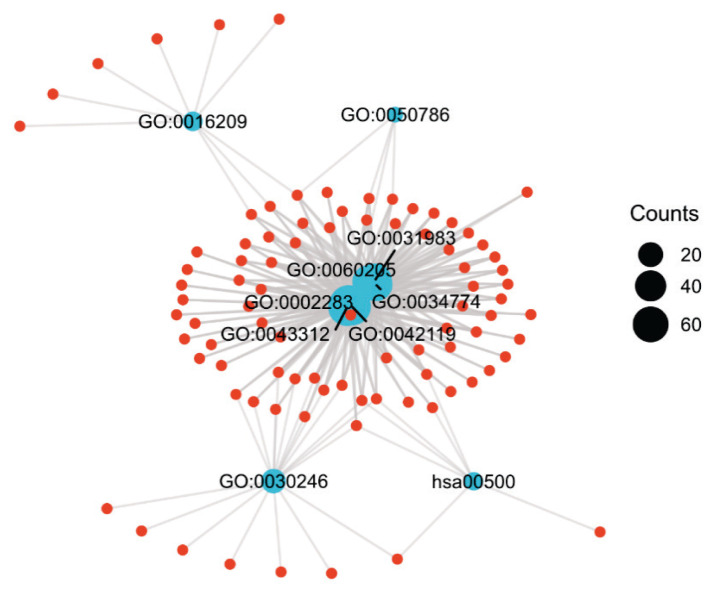
Functional clustering GO and KEGG visualization. Blue dots represent Ontology, red dots represent DEG, wires represent the relationship between them, and the size of the blue dots represents the number of DEG. DEG, differentially expressed gene.

Two hundred eighty-nine GO terms and one KEGG pathway exhibited enrichment that was statistically significant. The top three GO terms and the one identified KEGG pathway are shown in Supplementary Table 1, and the visualization network is shown in Fig. 3.

### Results of ferroptosis analysis

**Fig. 4:**
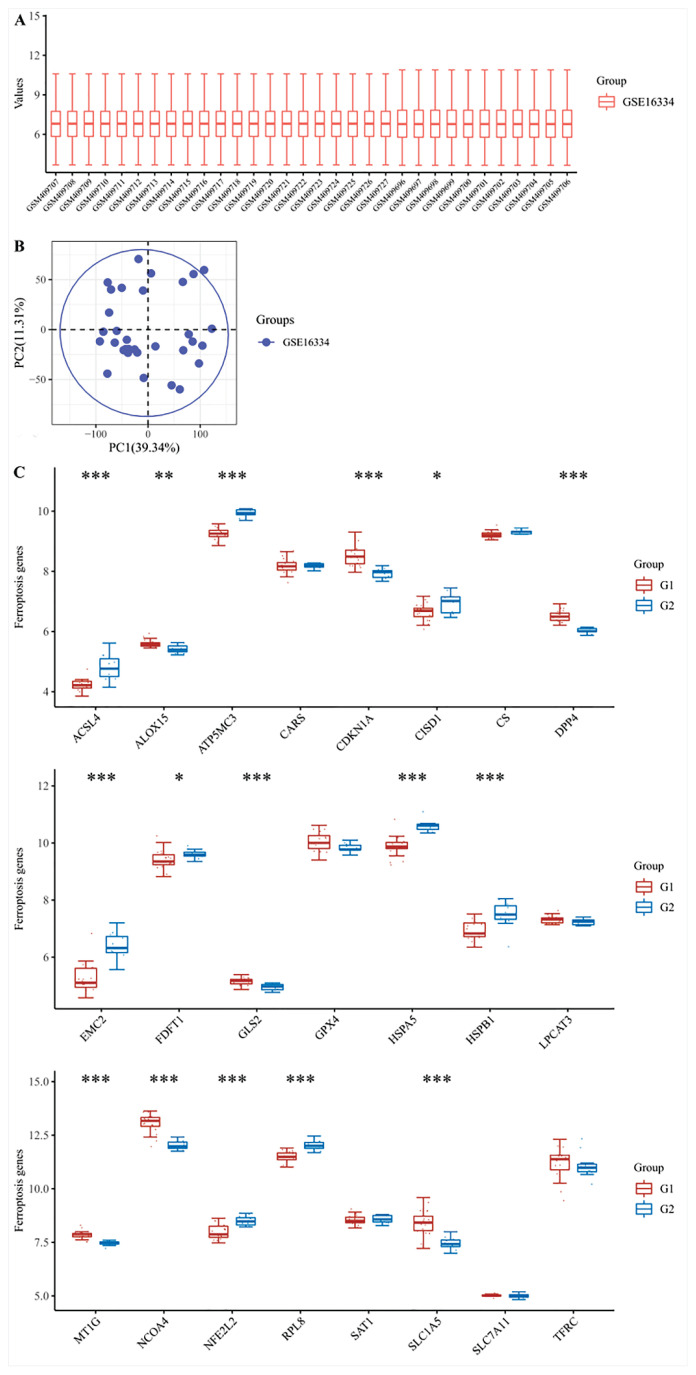
(**A**) The boxplot of the normalized data. Different colors represent different datasets. Rows represent samples, and columns represent the gene expression values in the samples. (**B**) PCA results before batch removal for multiple datasets. (**C**) The analysis result of ferroptosis-related genes. The expression distribution of ferroptosis in fanconi anemia group and normal group. The abscissa represents different ferroptosises, and the ordinate represents the expression distribution of gene, different colors represent different groups. *p < 0.05, **p < 0.01, ***p < 0.001, asterisks (*) stand for significance levels. The statis-tical difference of two groups was compared through the Wilcox test.

Sixteen genes related to ferroptosis were identified as statistically significant in the ferroptosis analysis, including *ACSL4, CDKN1A, CISD1, DPP4, ALOX15, ATP5MC3, EMC2, FDFT1, MT1G, NCOA4, NFE2L2, GLS2, HSPA5, SLC1A5, HSPB1 and RPL8*. The results are displayed in Fig. 4.

### Identification of critical FA genes through the PPI network

**Fig. 5:**
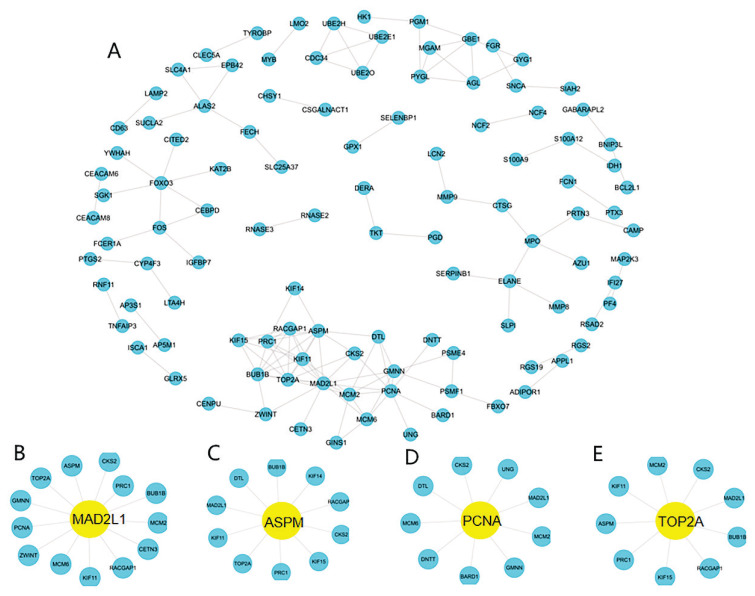
The protein-protein interaction (PPI) network for the DEGs. (**A**) PPI network for 109 DEGs, each round node represent a gene; (**B–E**) Sub-clusters of PPI network, the yellow round node indicated the key gene of sub-cluster.

The PPI network was used to develop a network for the 298 identified DEGs. Four significant sub-clusters were identified that exhibited sub-cluster scores of 12.347, 9.657, 8.615, and 8.611. The critical genes for each of the four subclusters were *MAD2L1, ASPM, PCNA*, and *TOP2A*, respectively (Fig. 5).

### Results of gene correlation analysis

**Fig. 6:**
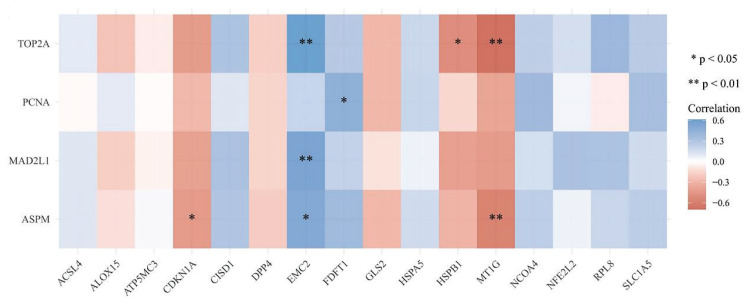
A heatmap of the correlation between multiple genes and multiple genes. The abscissa and ordinate represent genes, different colors represent different correlation coefficients (blue represents positive correlation whereas red represents negative correlation), the darker the color, the stronger the relation. Asterisks (*) stand for significance levels, ** for p < 0.01, * for p < 0.05.

The gene correlation analysis was performed between 16 ferroptosis-related genes (*ACSL4, CDKN1A, CISD1, DPP4, EMC2, ALOX15, ATP5MC3, HSPB1, MT1G, NCOA4, NFE2L2, FDFT1, RPL8, GLS2, HSPA5*, and *SLC1A5*) and essential genes associated with PPI (*MAD2L1, ASPM, PCNA*, and *TOP2A*). As a result, five statistically significant ferroptosis-related genes (critical genes of ferroptosis) were identified, which were *CDKN1A, EMC2, FDFT1, HSPB1*, and *MT1G* (Fig. 6).

### Validation of critical genes of ferroptosis by qPCR

The mRNA expression differences associated with these genes (*CDKN1A, EMC2, FDFT1, HSPB1*, and *MT1G*) between the normal and FA groups were significant (Supplementary Fig. 1).

### Diagnostic values of CDKN1A, EMC2, FDFT1, HSPB1, and MT1G in FA

The values associated with the diagnosis of FA for genes *CDKN1A, EMC2, FDFT1, HSPB1*, and *MT1G* were assessed. Initially, the GSE16334 dataset was utilized for training. The AUC values of the univariate ROC model for the five genes listed above were 0.965, 0.939, 0.745, 0.861, and 0.974, respectively. The AUC value of the joint model of the above genes was 1.00, indicating that these genes could be used to diagnose FA sufficiently (Supplementary Fig. 2A,B). Next, qRT-PCR was used to identify the differences in mRNA expression of the above genes for the verification of the results. Furthermore, the diagnostic value associated with the five genes corresponded well to results that were obtained with the GSE95095 dataset (Supplementary Fig. 2C,D). The AUC values obtained from the univariate ROC model of the above genes were 0.907, 0.640, 0.902, 0.840, and 0.929, respectively, and the AUC value of the joint model of these genes was 1.00.

### The Levels of mRNA expression for CDKN1A, EMC2, FDFT1, HSPB1, and MT1G in pan-cancer

The expression of mRNA of *CDKN1A, EMC2, FDFT1, HSPB1*, and *MT1G* in the TCGA and GTEx datasets were evaluated through the analysis of pan-cancer. The results that are shown in Supplementary Fig. 3 revealed that these five genes exhibited significant expression in BRCA, CSCC, LGG, and OV. In addition, *CDKN1A* and *EMC2* were significantly expressed in GBM, and *HNSC, FDFT1*, and *HSPB1* were significantly expressed in GBM.

### Prognostic values of CDKN1A, EMC2, FDFT1, HSPB1, and MT1G in pan-cancer

*CDKN1A, EMC2, FDFT1, HSPB1*, and *MT1G* were assessed for their ability to serve as prognostic indicators in pan-cancer by utilizing the TCGA database. We observed that elevated *CDKN1A* expression was significantly linked to decreased overall survival (OS) as well as to disease-specific survival (DSS) in LGG. It also was linked to shortened progress-free survival (PFS) in GBM (Supplementary Fig. 4A). Low *EMC2* expression was linked significantly to the shortened DSS and OS in BRCA and the shortened disease-free survival (DFS) in HNSC (Supplementary Fig. 4B). High expression of *FDFT1* was significantly linked with the lengthened OS and DSS in GBM and LGG and the lengthened PFS in LGG (Supplementary Fig. 4C). Elevated *HSPB1* expression was linked significantly to the shortened OS, DFS, PFS, and DSS in LGG and the lengthened DSS in HNSC (Supplementary Fig. 4D). *MT1G* was not significantly linked with prognosis in pan-cancer (Supplementary Fig. 4E).

## Discussion

More and more genes associated with FA have been identified. We discovered five ferroptosis-related genes, including *CDKN1A, EMC2, FDFT1, HSPB1*, and *MT1G* that might be associated with FA. The AUCs of these five ferroptosis genes alone for the diagnosis of FA ranged from 0.640 to 0.929, and the AUC reached 1.00 when the five genes were used in combination. Furthermore, the ferroptosis genes *CDKN1A, EMC2, FDFT1, HSPB1*, and *MT1G* were expressed in patients with GBM, LGG, HNSC, and BRCA. In addition, these genes were connected with OS, DFS, PFS, and DSS in individuals with these cancers.

Previous studies have reported that FA might be the result of an abnormality in one of the 22 FA/BRCA genes, which leads to DNA damage and repair defects, particularly the inability to repair chain cross-links [1,8]. The cancer-susceptibility characteristics of FA make the incidence of cancer in FA patients much higher than that seen in the general population [3].

Ferroptosis carries out essential functions in tumor development. Induction of ferroptosis can inhibit tumor growth, while ferroptosis injury may initiate inflammation-related immunosuppression in the microenvironment of the tumor, resulting in support for tumor growth [7]. There might be an unknown genetic association between ferroptosis and FA. In this investigation, differentially expressed genes between FA and control patients were analyzed, and four vital non-FA genes, *MAD2L1, ASPM, PCNA*, and *TOP2A*, were identified. In addition, 16 ferroptosis-related genes were identified when FA patients and control participants were compared. Gene correlation analysis indicated that five ferroptosis-related genes, including *CDKN1A, EMC2, FDFT1, HSPB1*, and *MT1G* were associated with four non-FA genes. The mRNA expression results confirmed the presence of significant expression differences in the five ferroptosis-related genes when FA patients were compared to the control participants. Song *et al*. found that the FA gene FANCD2 is associated with negative ferroptosis regulation through the regulation of iron metabolism involvement (e.g., *FTH1, TF, HSPB1, TFRC*, and *HAMP*) as well as the expression of lipid peroxidation protein genes [11]. Our study found an association between non-FA genes and ferroptosis genes in FA patients. Thus, these results are suggestive that a relationship exists between non-FA genes and ferroptosis genes.

Our results also showed the diagnostic value of five genes related to ferroptosis in FA patients as well as their value as prognostic indicators in multiple cancers. Diagnosis of FA is challenging due to the range of symptoms that can occur as well as the similarities that are observed between a range of other syndromes and FA [18,19]. FA also may be underdiagnosed [8,20]. FA has occasionally been observed in patients who develop abnormal cancers or toxicity after anticancer therapy, including myelodysplasia or a delay in the recovery of bone marrow function after chemotherapy [21,22]. Previous investigations concerning FA diagnoses have concentrated on FA-related genes [23,24], which might miss some patients diagnosed with FA based on other disease tests. Our results found that ferroptosis-related genes, including *CDKN1A, EMC2, FDFT1, HSPB1*, and *MT1G*, had good predictive outcomes for the diagnosis of FA. The AUC of the ferroptosis gene *MT1G* reached 0.929 for the diagnosis of FA, and the AUC was 1.00 when the five genes were utilized in combination.

The five genes related to ferroptosis might have critical roles in the discovery of FA patients in clinical practice. Expression of these genes that are related to ferroptosis and are identified in patients during genetic testing associated with other tumors or diseases might indicate a diagnosis of FA. The association between FA and ferroptosis-related genes has been reported in previous studies [11,25]. Ceccaldi *et al*. [25] reported the presence of hyperactivation of p53 in FA cells, which activated the cell cycle inhibition-related gene *CDKN1A* as a response to replication stress and DNA damage that was unresolved in patients with FA.

Song *et al*. [11] suggested that the FA-related gene *FANCD2* regulated the expression of genes that are involved in the metabolism of iron (*HSPB1*) as well as the peroxidation of lipids (*GPX4*). Also, these five ferroptosis-related genes have been demonstrated to be linked to the prognosis of multiple cancers. Our results indicated that elevated expression of *CDKN1A, HSPB1*, and *FDFT1* was linked to decreased OS and DSS in patients with LGG. Decreased *EMC2* expression was related to poorer OS and DSS in patients with BRCA. Monitoring the expression levels of FA-related genes is helpful for clinicians to identify high-risk patients as early as possible and make timely medical treatment plans to improve patient prognosis. Future research may develop drugs that block iron death to help hematopoietic stem cells proliferate and treat other bone marrow diseases, and may explore the related gene therapy to treat diseases.

This study investigated the predictive value of five ferroptosis-related genes, including *CDKN1A, EMC2, FDFT1, HSPB1*, and *MT1G*, in FA diagnosis. We also assessed the expression of these five genes associated with ferroptosis and evaluated their prognostic value in multiple cancers. However, limitations associated with this investigation need to be considered. First, the predictive value of ferroptosis-related genes in FA patients was assessed based on 11 normal participants and 21 FA patients, and future studies might require a larger number of samples. Second, the ability to obtain detailed patient information was somewhat limited in this study. Therefore, the relationship between genes associated with ferroptosis and the prognosis of multiple cancers was not adjusted for a range of variables, and additional confounding factors may have affected the results. Third, the functions and mechanisms of action of the five ferroptosis-related genes, including *CDKN1A, EMC2, FDFT1, HSPB1*, and *MT1G* in FA, have not been clarified.

## Conclusions

This study analyzed the possible genetic association between FA and ferroptosis. Five ferroptosis genes were identified, including *CDKN1A, EMC2, FDFT1, HSPB1*, and *MT1G*, which were associated with non-FA genes. The five ferroptosis-related genes showed good predictive value for the diagnosis of FA. The AUC of one gene for predicting FA was 0.929, and the AUC of the combination of the five genes reached 1.00. In addition, the expressions of *CDKN1A, EMC2, FDFT1*, and *HSPB1* were associated with the prognosis of multiple cancers. Future studies should explore the functions and mechanisms of actions of the five ferroptosis-related genes *CDKN1A, EMC2, FDFT1, HSPB1*, and *MT1G* in FA.

## Supplementary Information
