# Supplementary material for: Key Ferroptosis Genes and their Predictive and Diagnostic Value in Fanconi Anemia
Source: Physiol Res. 2025 Apr 1;74(2):275–85. doi: 10.33549/physiolres.935383 (PMC12148158; doi:10.33549/physiolres.935383)
Supplement: Supplementary file 1 [file PR74_275_Supplemental_data.pdf]

Supplementary Table 1. Top 3 gene ontology (GO) terms and a pathway (in the order of increasing P.adjust). BP, biological pathways; CC, cellular components; MF, molecular function.

**Ontology**	**ID**	**Description**	**p-value**	**adjust p-value**	**q-value**	**rank**
BP	GO:0043312	neutrophil degranulation	5.13e-50	1.51e-46	1.26e-46	1
BP	GO:0002283	neutrophil activation involved in immune response	8.00e-50	1.51e-46	1.26e-46	2
BP	GO:0042119	neutrophil activation	3.44e-49	3.74e-46	3.12e-46	3
CC	GO:0034774	secretory granule lumen	3.83e-35	1.54e-32	1.37e-32	1
CC	GO:0060205	cytoplasmic vesicle lumen	4.77e-34	7.40e-32	6.57e-32	2
CC	GO:0031983	vesicle lumen	5.51e-34	7.40e-32	6.57e-32	3
MF	GO:0030246	carbohydrate binding	1.38e-07	7.85e-05	6.78e-05	1
MF	GO:0016209	antioxidant activity	1.25e-05	0.003	0.003	2
MF	GO:0050786	RAGE receptor binding	2.22e-05	0.003	0.003	3
KEGG	hsa00500	Starch and sucrose metabolism	3.52e-06	8.21e-04	7.97e-04	1

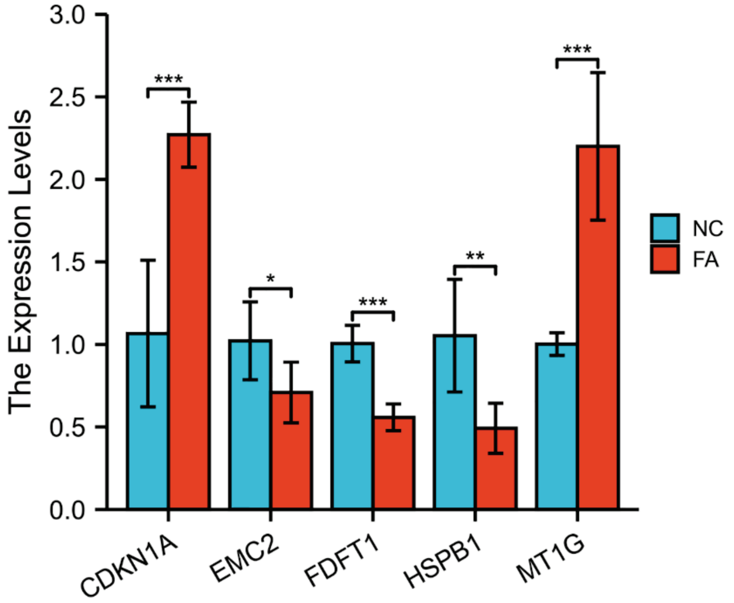

Supplementary Figure 1. Verification of mRNA expression. Gene expression in normal (n=5) and FA (n=5) tissues was evaluated by qPCR and normalised against glyceraldehyde-3-phosphate dehydrogenase (GAPDH) expression. *p < 0.05, **p < 0.01, ***p < 0.001.

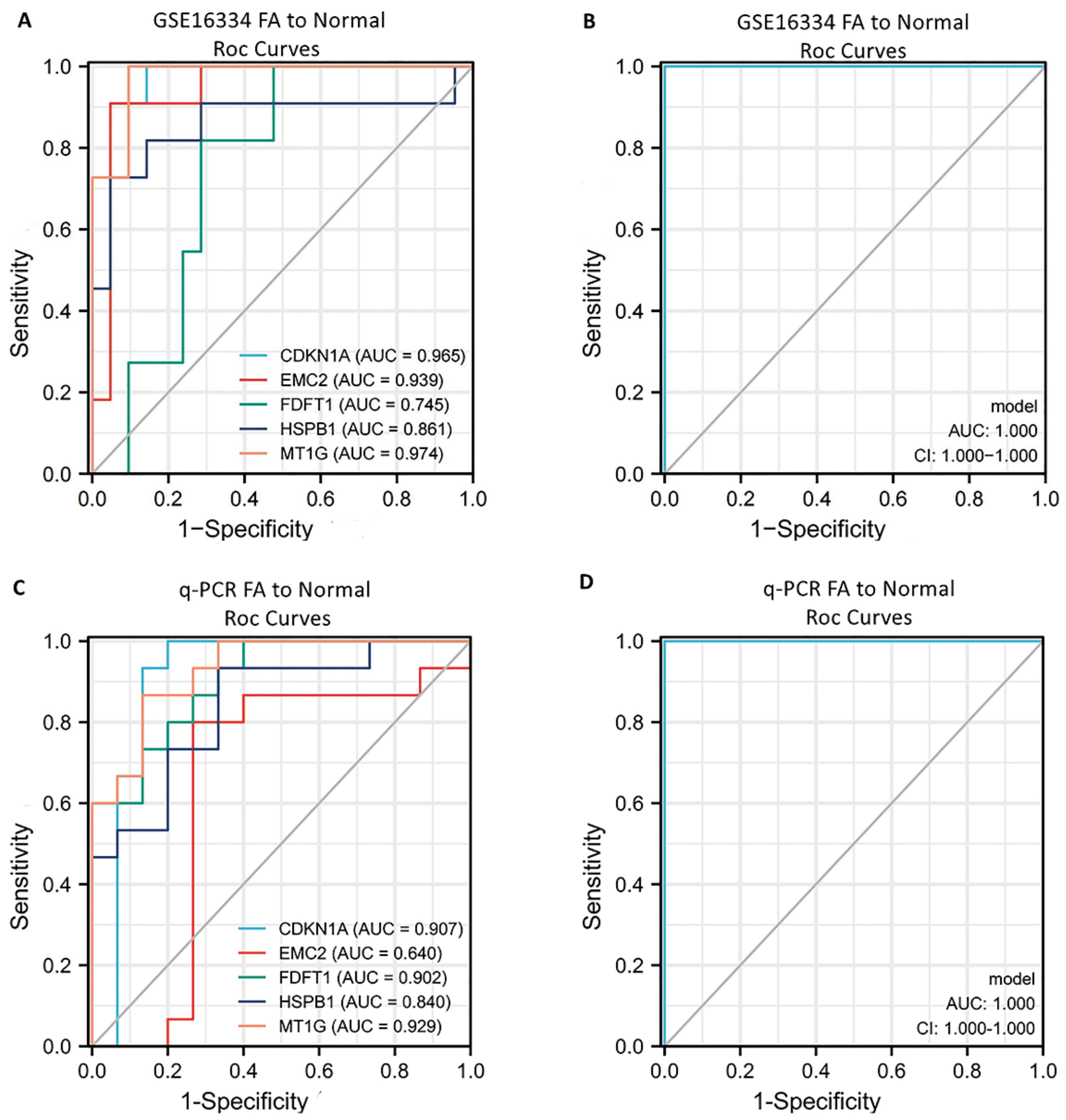

Supplementary Figure 2. Diagnostic values of CDKN1A, EMC2, FDFT1,HSPB1 and MT1G in FA. (**A**) univariate Roc model(GSE16334); (**B**) joint Roc model(GSE16334);(**C**) univariate Roc model(q-PCR); (**D**) joint Roc model(q-PCR).

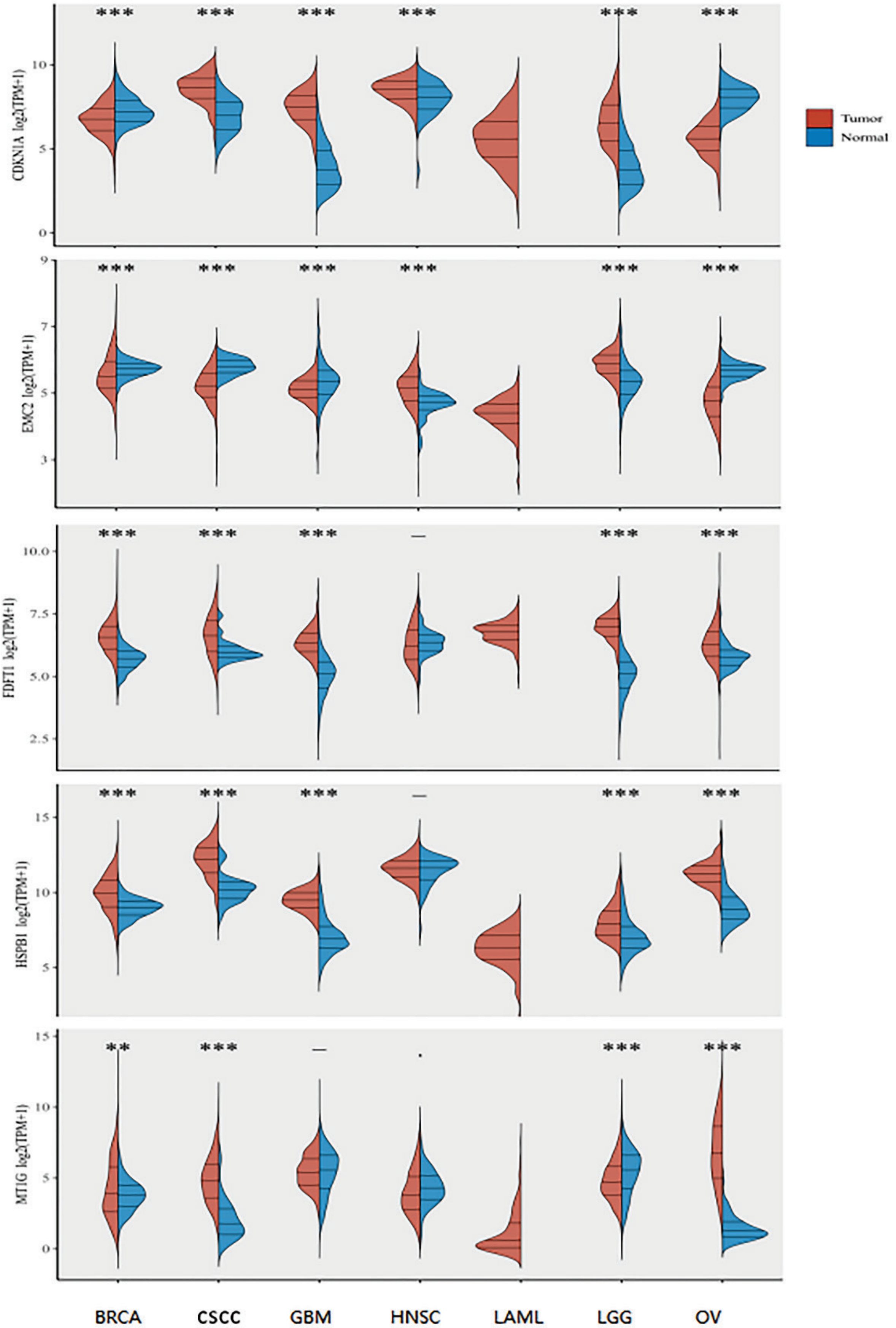

Supplementary Figure 3. Expression patterns of CDKN1A, EMC2, FDFT1, HSPB1 and MT1G in pan-cancer. The expression patterns of CDKN1A, EMC2, FDFT1, HSPB1 and MT1G were analyzed by using TCGA and GTEx data. *p < 0.05, **p < 0.01,***p < 0.001.

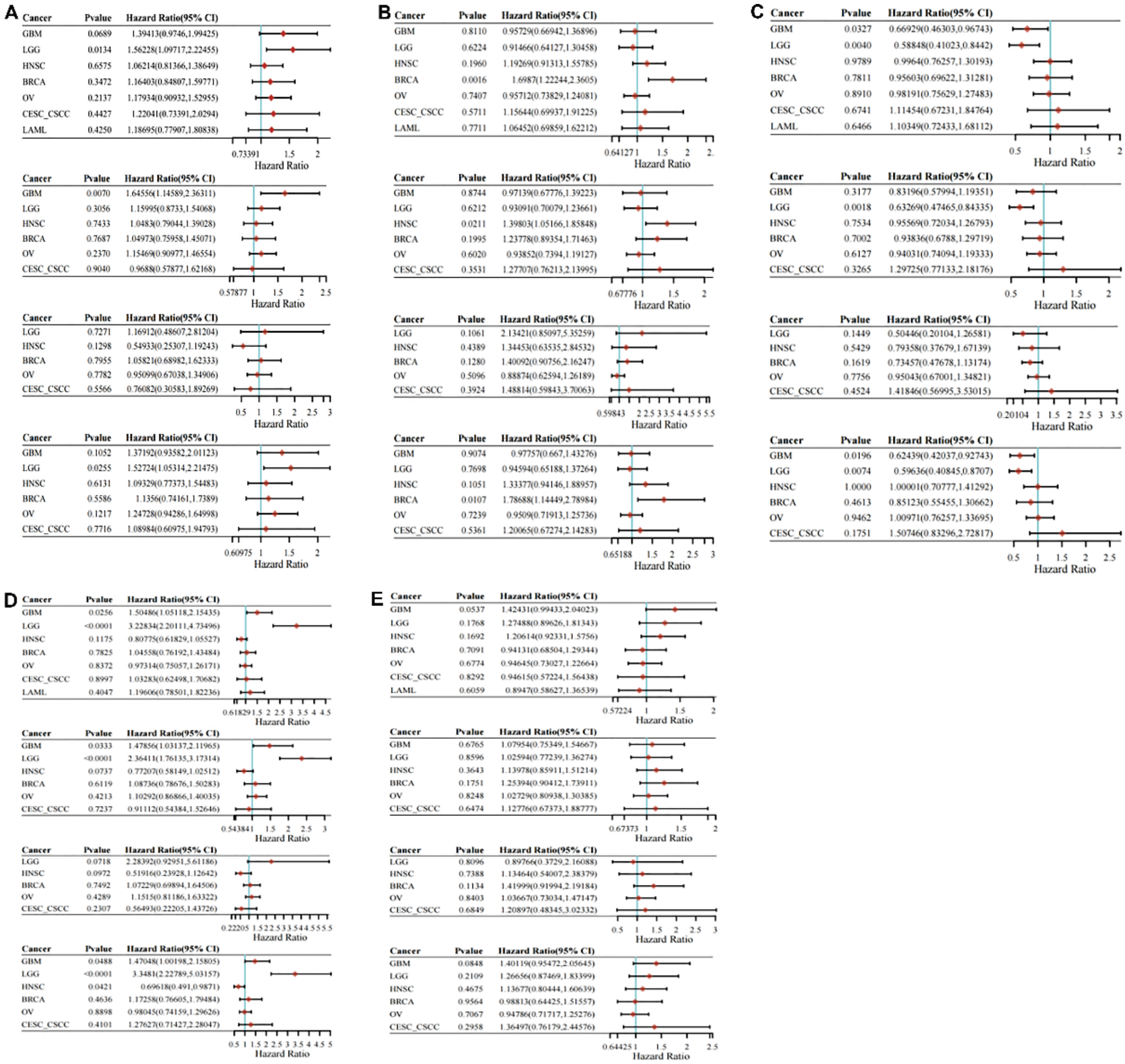

Supplementary Figure 4. Prognostic roles of CDKN1A, EMC2, FDFT1, HSPB1 and MT1G in pan-cancer. (**A-E**) The p-value, risk coefficient (HR) and confidence interval of CDKN1A, EMC2, FDFT1, HSPB1 and MT1G in multiple tumours are analyzed by by using TCGA database.
